# Anterior Cruciate Ligament Reconstruction with Semitendinosus Tendon Autograft among Paramilitary Patients Undergoing Arthroscopic Surgery in a Tertiary Care Centre

**DOI:** 10.31729/jnma.8417

**Published:** 2024-01-31

**Authors:** Sailendra Kumar Duwal Shrestha, Prabin Nepal, Umash Karki, Netra Bahadur Karki, Samir KC, Rojan Tamrakar, Kumar Shrestha, Pramod Joshi

**Affiliations:** 1 Department of Orthopedic and Trauma Services, Nepal Armed Police Force Hospital, Balambu, Kathmandu, Nepal; 2 Department of Orthopedics, National Trauma Center, Mahankal, Kathmandu, Nepal

**Keywords:** *anterior cruciate ligament*, *grafts*, *semitendinosus tendon*

## Abstract

**Introduction:**

In orthopaedic practice, injuries to the anterior cruciate ligaments occur almost on an epidemic scale, yet it continues to be of interest in orthopaedic surgery whether semitendinosus or gracilis hamstring autografts can be used for better anterior cruciate ligament reconstruction. This study aimed to determine the prevalence of anterior cruciate ligament reconstruction using semitendinosus tendon autografts among paramilitary patients undergoing arthroscopic surgery in a tertiary care centre.

**Methods:**

This descriptive cross-sectional study was conducted among paramilitary individuals who had knee injuries and were admitted between 6 February 2020 and 26 January 2022 for arthroscopic surgery after obtaining ethical approval from the Institutional Review Committee. Demographic details and the mode of injury were obtained from the patients. The treating orthopaedic surgeons evaluated the pre- and post-analysis Lysholm Knee Score and Lysholm Knee Scale based on the patient's response. A convenience sampling method was used. The point estimate was calculated at a 95% Confidence Interval.

**Results:**

Among 166 patients, anterior cruciate ligament reconstruction using a semitendinosus tendon autograft was done in 58 (34.94%) (27.69-42.19, 95% Confidence Interval). Most of the patients in the pre-analysis had mild/periodic limp issues 52 (89.66%), followed by instability during athletics or other severe exertion 43 (74.14%).

**Conclusions:**

The prevalence of anterior cruciate ligament injuries in our study is higher than other studies done in similar settings.

## INTRODUCTION

The paramilitary community represents a unique population that is exposed to extreme physical stress, including long-distance running and weight-bearing exercises.^1^ The effects of this type of practice can lead to joint overuse injuries, such as an injured anterior cruciate ligament (ACL) sustained from strenous exercise or excessive stress placed on the knee (>1725 N), which exceeds the ACL's ability to withstand and heal.^2^

ACL reconstruction is an arthroscopic procedure that is widely performed worldwide, often with autografts (bone-patellar tendon and hamstring) and allografts.^3^ Hamstring autografts using gracilis tendons or semitendinosus tendons are generally preferred at present,^4^ but there are still discrepancies among orthopaedic specialists regarding the preparation technique (scar formation, operating time, infection risk) and the graft preference (two or four strands, diameter, and length).^5^

The aim of this study was to determine the prevalence of ACL reconstruction with semitendinosus tendon autograft (STA) among paramilitary patients undergoing arthroscopic surgery in a tertiary care centre.

## METHODS

A descriptive cross-sectional study was conducted among the paramilitary patients who had undergone arthroscopic surgery from 6 February 2020 and 26 January 2022 in the Nepal Armed Police Force Hospital (NAPFH) Balambu, Kathmandu, Nepal. Ethical approval was obtained from the Nepal Health Research Council (Reference number: 2390). The paramilitary patients, aged 18 to 50 years, who attended the Department of Orthopedics due to knee injuries, indicated for arthroscopic surgery, were included in the study. Patients who did not give consent were excluded from the study. Convenience sampling was done. The sample size was calculated using the following formula.


$$\begin{array}{ll} \text{n} & ={\text{Z}}^{2}\times \frac{\text{p}\times \text{q}}{{\text{e}}^{\text{2}}}\\ & =1.96^{2}\times \frac{0.50\times 0.50}{0.08^{2}}\\ & =151 \end{array}$$

Where,

n = minimum required sample size
z = 1.96 at 95 % Confidence Interval (CI)
p = prevalence taken as 50% for maximum sample size calculation
q = 1-p
e = margin of error, 8%

The calculated minimum sample size was 151. However, we have included 166 patients.

Each patient underwent a series of clinical tests, such as the Lachman test, anterior drawer test, pivot shift test, and an MRI (of the affected knee) to confirm a complete tear of the ACL. An oblique incision for graft retrieval on the anteromedial aspect of the tibia was performed. The semitendinosus tendon was hooked and released from all tendinous ramifications following the incision of the sartorius fascia in an oblique fashion parallel to the direction of the pes tendons. To maximize the length of the isolated semitendinosus tendon, the tibial site attachment was severed as close to the bone as possible while taking every precaution to avoid damaging the additional conjoint tendon. The semitendinosus graft ends were stitched together and then folded into four strands in a quadrupled pattern. The patients were then treated with standard knee arthroscopy portals and a single bundle ACL reconstruction with semitendinosus autograft. Postoperatively, the patients were put on an ACL rehabilitation protocol that included isometric quadriceps exercises, crutches, and a knee brace for the first 2 weeks of walking. After 3 weeks, a normal gait pattern without crutches and knee brace was encouraged, and non-contact sports were only permitted for at least 4 months.

Patients' details were acquired from the NAPFH nondigital database. Demographic details (age, sex, occupation) and mode of injury were obtained from the study patients. The functional status of the patient was assessed by using parameters such as pain, limping, locking, instability, swelling, stair climbing, squatting, and the need for support. The Lysholm Knee Score was provided both pre-operatively and post-operatively (1 year later). Similarly, the functional outcome was graded as excellent, good, fair, or poor.^6^ The treating orthopaedic surgeon based on the patient's response, gave scores and scales to each patient.

The study data were entered in Microsoft Excel 2010. The statistical analyses were performed in IBM SPSS Statistics version 17.0. The point estimate was calculated at a 95% CI.

## RESULTS

Among 166 patients, ACL injuries treated using a semitendinosus tendon autograft were 58 (34.94%) (27.69-42.19, 95% CI). The majority of the patients belonged to the age group 26-30 years 17 (29.31%) (Table 1).

**Table 1:** Age-wise distribution of patients (n= 58).

Age group (years)	n (%)
18-20	4 (6.90)
21-25	16 (27.59)
26-30	17 (29.31)
31-35	16 (27.59)
36-40	5 (8.62)

**Figure 1:**
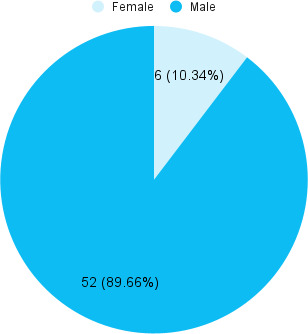
Gender-wise distribution (n = 58).

A total of 52 (89.66%) were males (Figure 1)

**Figure 2:**
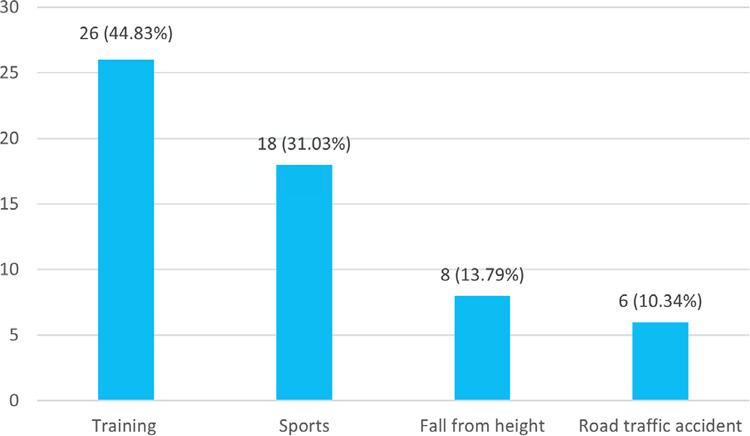
Modes of injuries (n = 58).

Among them, 26 (44.83%) were due to training and 18 (31.03%) were due to sports (Figure 2).

Right knee 32 (55.17%) was injured more (Table 2).

**Table 2:** Laterality of the injured knee (n = 58).

Laterality	n (%)
Left	26 (44.83)
Right	32 (55.17)

In the pre-analysis, 52 (89.66%) patients had limps, 34 (58.62%) had occasional pains, 43 (74.14%) had instability rarely during athletics and other severe exertion, and 37 (63.79%) had swelling on exertion. Similarly, after 1 year, at least 54 (93%) patients had none of these symptoms and 3 (5.17%) had impairments in both squatting and climbing stairs (Table 3).

**Table 3:** Lysholm Knee Score parameters among the study participants (n = 58).

Parameters (score)	Pre-operative n (%)	Post-operative n (%)
**Limp**		
Severe or Constant (0)	3 (5.17)	-
Slight/periodical (3)	52 (89.66)	-
None (5)	3 (5.17)	58 (100)
**Pain**		
Marked on or after walking more than 2 km (10)	10 (17.24)	-
Marked during severe exertion (15)	14 (24.14)	1 (1.72)
Inconstant and slight during exertion (20)	34 (58.62)	3 (5.17)
None (25)	-	54 (93.10)
**Locking**		
Catching sensation but no locking (10)	8 (13.79)	7 (12.07)
No locking and no catching sensation (15)	50 (86.21)	51 (87.93)
**Stairs**		
No problem (10)	3 (5.17)	55 (94.83)
One step at a time (2)	18 (31.03)	-
Slightly impaired (6)	37 (63.79)	3 (5.17)
**Support**		
None (5)	58 (100)	58 (100)
**Instability**		
Occasionally on daily activities (10)	1 (1.72)	-
Frequently during athletics and other severe exertion (15)	9 (15.52)	-
Rarely during athletics and other severe exertion (20)	43 (74.14)	3 (5.17)
Never giving way (25)	3 (5.17)	55 (94.83)
Occasionally on daily activities (5)	2 (3.45)	-
**Swelling**		
None (10)	3 (5.17)	58 (100)
On ordinary exertion (2)	18 (31.03)	-
On severe exertion (6)	37 (63.79)	-
**Squatting**		
Slightly impaired (4)	50 (86.21)	3 (5.17)
No problem (5)	8 (13.79)	55 (94.83)

A total of 45 (77.59%) showed a fair Lysholm Knee Scale scale in pre-analysis, while 52 (89.66%) showed an excellent scale in post-analysis (Table 4).

**Table 4:** Grading of functional outcomes (n= 58).

Scales	Pre-operative n(%)	Post-operative n(%)
Poor (<65)	10 (17.24)	-
Fair (65-83)	45 (77.59)	6 (10.34)
Good (84-90)	1 (1.72)	-
Excellent (>90)	2 (3.45)	52 (89.66)

## DISCUSSION

The prevalence in our study was found to be 58 (34.94%). In another study, the prevalence of ACL injury was found to be 14.7%.^7^ Similarly, in a previous study, 67.52% of the participants had ACL tears.^8^ In 52.11% of cases, the semitendinosus tendon was used for anterior cruciate ligament reconstruction in the form of a tripled or quadrupled graft as reported by another study.^9^

In this study, 52 (89.66%) males had an ACL injury about nine times more frequently than females 6 (10.34%). An insignificant gender difference in overall risk was found in another study.^10,11^ The higher percentage of men recruited into the paramilitary force or their propensity to participate in sports activities could both be contributing factors to the higher incidence of ACL injuries in males in this study.

Military training was the major cause of ACL injuries 26 (44.83%), followed by sport-related activities 18 (31.03%), falls from heights 8 (13.79%), and automobile accidents 6 (10.34%). A study found that a higher incidence of ACL injuries was associated with sporting activities (53.26%), while the remainder was associated with non-sporting activities (46.74%)^12^ which is inconsistent with the results of this study. These injuries frequently happen as non-contact injuries, which mostly happen during activities requiring rapid deceleration, which causes the knee to extend excessively, and during the knee's pivoting, which happens when rapidly changing directions.^13,14^

The post-analysis revealed that 84.45% of patients with an excellent Lysholm Knee Scale had previously been classified as poor (17.24%), fair (7.59%), or good (1.72%) in the pre-analysis. An improved Lysholm knee score was also reported in the studies done in similar settings.^15^ Nonetheless, we support the use of the semitendinosus tendon alone as a suitable graft option if the tendon is of adequate length.

The major limitation of this research is its smaller sample size, which was drawn from only one tertiary care hospital and might not be entirely representative of the general population.

## CONCLUSIONS

The prevalence of ACL injuries in our study is higher than in other studies done in similar settings. A large multicenter study is required to evaluate the effectiveness of treatment outcomes using semitendinosus tendon autograft with other types of graft in ACL injuries.
